# Habitual Dietary Collagen Intake Is Lower in Females and Older Irish Adults Compared with Younger Males

**DOI:** 10.1016/j.tjnut.2025.03.002

**Published:** 2025-03-08

**Authors:** Christopher D Nulty, Janette Walton, Robert M Erskine

**Affiliations:** 1 School of Sport and Exercise Sciences, Liverpool John Moores University, Liverpool, United Kingdom; 2 Department of Health and Sport Science, South East Technological University, Carlow, Ireland; 3 School of Food and Nutritional Sciences, University College Cork, Cork, Republic of Ireland; 4 Department of Biological Sciences, Munster Technological University, Cork, Republic of Ireland; 5 Institute of Sport, Exercise and Health, University College London, London, United Kingdom

**Keywords:** glycine, proline, hydroxyproline, vitamin C, protein

## Abstract

**Background:**

Collagen ingestion reportedly benefits connective tissues, such as skin, bone, muscle, tendon, and ligament. However, the quantity of collagen intake in the diet of European adults is unknown.

**Objectives:**

This study aims to investigate collagen intake in the habitual diets of Irish adults, and whether it differed according to sex and/or age.

**Methods:**

We conducted secondary analysis of the Irish National Adult Nutrition Survey, which assessed typical dietary intake using a 4-d food diary in 1500 adults, aged 18–90 y. We categorized participants into 3 age groups: young (18–39 y, *n* = 630), middle-aged (40–64 y, *n* = 644), and older (≥65 y, *n* = 226) adults. Collagen composition of each individual food item in the database was determined by applying a percentage collagen value from analytical sources, allowing computation of collagen mean daily intake (MDI), collagen MDI relative to body mass, and collagen/total protein MDI. Differences in intakes between age groups and sexes were evaluated using physical activity level as a covariate.

**Results:**

Collagen MDI for the entire population was 3.2 ± 2.0 g/d, representing 3.6% ± 1.9% total protein intake. Males had higher absolute and relative collagen MDI than females, regardless of age (4.0 ± 2.1 g/d compared with 2.3 ± 1.4 g/d, *P* < 0.001), whereas older adults had lower absolute collagen MDI than middle-aged adults (2.9 ± 1.8 g/d compared with 3.3 ± 2.0 g/d, *P* = 0.021).

**Conclusions:**

Collagen intake in the Irish adult population was considered low (relative to total protein intake and to dose–response studies), particularly in females and older individuals. Increasing daily collagen intake may therefore be warranted to optimize the health of collagen-rich tissues.

## Introduction

Collagen is the most abundant protein in the human body, found in the extracellular matrix (ECM) of connective tissue (for example, in skin, bones, tendon, ligament, and cartilage). Collagen is synthesized by fibroblasts through a process that involves the transcription of collagen genes, translation into preprocollagen, and extensive post-translational modifications, with vitamin C being a crucial cofactor for proper folding into a triple-helix structure [1]. Collagen synthesis decreases with age, whereas collagen degradation increases simultaneously, leading to reduced dermal volume and elasticity, which can be observed as increased skin wrinkles and slower cutaneous wound healing [2,3]. Moreover, collagen loss with age is associated with bone loss and increased tendon compliance, which are both risk factors for higher fracture rates and fall injuries in older adults [4,5].

Dietary collagen is rich in glycine, proline, and hydroxyproline, with the latter being exclusive to collagen-containing foods. Although collagen can be synthesized endogenously, dietary intake is required to maintain sufficiently high levels of these amino acids, particularly glycine, which may be conditionally essential in humans [6,7]. For ingested collagen to contribute to systemic collagen turnover, it must undergo enzymatic hydrolysis in the gastrointestinal tract, being digested into peptides and free amino acids before being absorbed into the bloodstream [8]. In recent years, there has been significant interest in the effects of collagen intake, with and without exercise, on skin appearance, wound healing, joint pain, exercise recovery, body composition, sleep quality, and muscle and tendon function [[9], [10], [11], [12]]. Medium-to-long-term supplementation with 2.5–12 g hydrolyzed collagen (HC) appears to have positive effects on skin aging by increasing elasticity and hydration, and reducing the appearance of wrinkles in adult females aged 20–70 y [13]. Additionally, collagen ingestion combined with acute skipping or resistance exercise has been associated with increased systemic markers of collagen synthesis, with doses ranging between 5 and 30 g collagen in young, healthy, recreationally trained males and females [[14], [15], [16]]. Moreover, 15–30 g daily HC supplementation in conjunction with chronic exercise is reported to improve fat-free mass, tendon morphology, and markers of strength in both athletic and untrained adults [17].

These findings indicate positive effects of increasing dietary collagen intake; however, there is no established recommended daily allowance for collagen, and we cannot assume the dietary collagen requirements of adults are the same across the age range and between sexes. First, this is because aging is associated with collagen loss, especially after 40 y of age [18,19]. Second, connective tissues, comprising mostly collagen, such as ligament, tendon, and bone, all contain estrogen receptors [[20], [21], [22]], resulting in sex-specific collagen turnover [23]. Thus, before making recommendations on increasing collagen intake (either via dietary food or supplementation), the amount of collagen ingested in the habitual diets of both males and females across the age range must be documented.

Despite the clear interest in collagen supplements in areas of dermatology, musculoskeletal health, aging, and sports performance, only one study, to our knowledge, has attempted to estimate collagen intake in habitual diets. Paul et al. [24] estimated that the average daily collagen protein consumption in the “standard American diet” to be either 3 or 23 g/d (based on whether individuals were low or high consumers of processed meat), which was derived from the NHANES 2001–2004. However, estimates of collagen in food items were unjustifiably averaged across a limited number of food items (for example, beef, pork, veal, lamb, and game), which likely confounded the estimations of collagen intake, thus potentially providing erroneous conclusions.

The National Adult Nutrition Survey (NANS) investigated habitual food and beverage consumption in 1500 adults in Ireland between 2008 and 2010. By conducting a secondary analysis of NANS, we determined the collagen composition of each individual food item in the database by applying a percentage collagen value from analytical sources, allowing computation of collagen mean daily intake (MDI), relative MDI (g/kg), and collagen/total protein MDI (%). We categorized participants into young, middle-aged and older adults, and we compared males with females. Thus, the objective of our study was to determine the habitual dietary collagen intake of Irish adults, stratified by age group and sex, using detailed collagen content data from specific food items. In addition, because of vitamin C being an essential component for collagen synthesis [1], we investigated vitamin C intake according to age and sex. Finally, as increased physical activity (PA) is associated with a higher metabolic rate and greater energy intake [25], we used habitual PA as a covariate in our dietary analyses. Additionally, habitual PA is positively associated with protein intake in older adults across multiple populations [26], suggesting a potential link between PA levels and collagen intake, given that collagen is a specific source of dietary protein. Based on the higher habitual protein intake in younger males compared with older males and females [27], we hypothesized that absolute and relative collagen MDI would be lower in older and middle-aged individuals compared with young adults, and lower in females compared with males.

## Methods

### Population

The current study is a secondary analysis of a cross-sectional food consumption survey among Irish adults (described in detail elsewhere [27]). The original survey was the Irish NANS, conducted by the Irish Universities Nutrition Alliance (IUNA; www.iuna.net). This survey included 1500 free-living adults aged 18–90 y (740 males and 760 females) residing in the Republic of Ireland between 2008 and 2010. Ethical approval was granted by the University College Cork Clinical Research Ethics Committee of the Cork Teaching Hospitals and the Human Ethics Research Committee of University College Dublin. All participants provided written consent in line with the Declaration of Helsinki. Participants were randomly chosen from a database of names and addresses provided by Data Ireland (National Postal Service). An invitation letter and participant information sheet were sent to the homes of potential participants. Participants were excluded if they were pregnant or lactating or were unable to complete the survey because of disability. The survey achieved a response rate of 59.6%, and the final sample was demographically representative of the Irish population in terms of sex, age, location, social class, and geographical distribution according to the 2006 Irish census. The sample size has previously been demonstrated to be sufficient for detecting dietary intake differences by sex and age in recent secondary analyses of this dataset [27].

### Dietary assessment

A 4-d food diary (detailed at the product brand level where possible) was employed to record food, beverage, and supplement intake. Participants were required to include ≥1 weekend day in their recordings. Researchers visited participants’ homes 3 times during the 4-d period: the first visit demonstrated how to use a food weighing scales and maintain the food diary; the second visit, 24–36 h into the recording process, reviewed the diary entries; and the final visit, 1–2 d after the recording period, reviewed the last entries and collected the diary. Food and beverage consumption was quantified using food weighing scales where possible. However, for items that were not weighed, portion sizes were estimated using a photographic food atlas, a food portion size guide, household measurements, manufacturer weights, the IUNA weight guide, and researcher estimates. Nutrient intakes were estimated using Weighed Intake Software Program, version 3.0 (Tinuviel Software), based on data from McCance and Widdowson’s “The Composition of Foods,” 5th and 6th editions and their associated supplementary volumes [[28], [29], [30]]. Dietary intake was averaged across the 4 d to provide MDI for all nutrients of interest.

### Calculation of collagen composition

The collagen content of food items was determined by applying a percentage weighting from estimated typical values. A database [Statistical Package for the Social Sciences (SPSS) v. 29, IBM] was created containing all of the 2552 NANS foods consumed, including recipes. Second, this database was examined on a food code-by-food code basis, and each food code was assigned a collagen concentration based on analytical data and other published data sources, which are presented in Supplemental Table 1 [[31], [32], [33], [34], [35], [36], [37], [38], [39], [40], [41], [42], [43], [44]]. In total, 736 foods were identified to contain collagen. If a food item contained a meat mixture, that is, >1 meat cut or meat source, then the total collagen for that item was calculated using the following equation, which was modifiable to include additional ingredients as required [31]:

$$\frac{\left(A\times a\right)+\left(B\times b\right)+\left(C\times c\right)\ldots }{100}$$

where *A*, *B*, *C*, etc., represent the percentage of each meat cut present, and *a*, *b*, *c*, etc., represent the percentage of collagen in each meat cut. For example, an item containing 15% beef brisket lean (2.56% collagen), and 3% beef fat (5.76% collagen) would be calculated as:

$$\frac{\left(15\times 2.56\right)+\left(3\times 5.76\right)}{100}=\frac{38.4+17.28}{100}=0.56\%\;collagen$$

Collagen values for foods that were prepared whole but contained inedible portions, such as bone, were then multiplied by their edible conversion factor from the composition of foods integrated dataset [45]. For database items where the food label was not available, or the item was a meal/multi-ingredient item, for example, beef lasagna, or chicken korma, the percentage of foods containing collagen was determined by dividing the mass (g) of each respective food containing collagen in the recipe by the sum of the mass (g) of all items in the recipe expressed as a percentage of total mass. Where mixed foods were sandwiches without precise quantities of the constituents, the recipe from the University of London survey of commercial sandwiches was used for all calculations [46]. Finally, where the collagen content of meat was presented as a percentage of total protein content, rather than a percentage of total weight (for example, anchovies, bovine liver), the known total protein was multiplied by the collagen percentage to determine the collagen value for that food item.

### PA levels

Participants completed a validated PA questionnaire [European Prospective Investigation into Cancer Physical Activity Questionnaire (EPAQ2)] [47] to estimate habitual levels of PA. The questionnaire comprised 3 sections: activity at *1*) home, *2*) work, and *3*) recreation. To estimate participants’ metabolic equivalent of the task (MET) values, we used the EPAQ2 responses to calculate the average MET hours spent per week. MET values were assigned to various activities based on established MET values for each type of activity. The total weekly MET hours were then calculated by summing the MET hours from all 3 activity domains (home, work, and recreation). Participants were subsequently categorized into 3 activity levels based on their total weekly MET hours [48]: low activity: <7.5 MET h/wk; moderate activity: ≥ 7.5 to 15 MET h/wk; high activity: >15 MET h/wk.

### Secondary data analysis

Data were analyzed using the SPSS (v. 29, IBM) and reported as mean ± SD, with significance accepted at *P* < 0.05. The following new variables were computed and used for the analysis of collagen intake: absolute collagen MDI in grams; relative collagen MDI in g/kg (collagen MDI relative to body mass); and collagen/total protein MDI (%) (collagen MDI expressed as a percentage of total protein MDI).

To examine the effects of age group and sex on collagen intake, participants were assigned to 1 of 2 categories for sex (male or female), and 1 of 3 categories for age: *1*) young (18–39 y, male, *n* = 331; female, *n* = 299); *2*) middle-aged (40–64 y, male, *n* = 303; female, *n* = 341); and *3*) older (≥65 y, male, *n* = 106; female, *n* = 120). Data were assessed for normal distribution using visual inspection of Q–Q plots. Most nutrient intake variables approximated normality despite slight tail deviations in Q–Q plots (Supplemental Figure 1), typical of dietary data, and did not require transformation. However, protein intake, and correspondingly, collagen intake, exhibited slightly greater tail deviations in their respective Q–Q plots (Supplemental Figure 1). To ensure robustness, protein and collagen intake data were log-transformed, and the analyses were repeated. The results of these transformed analyses were consistent with those using the nontransformed data, suggesting that the observed effects were not influenced by these deviations. Chi-square tests of independence were performed to examine the association between age group, sex and PA levels. Differences in nutritional intake (that is, energy, protein, carbohydrate, fat, collagen, and vitamin C) between age group and sex were evaluated using one-way analysis of covariance (ANCOVA), with PA level category (low, moderate, high) incorporated as a covariate. Bonferroni adjustment was used for post-hoc comparisons. Partial eta squared (*η*_*p*_^2^) was reported as an estimate of effect size for ANCOVA main effects and interaction effects. The thresholds of *η*_*p*_^2^ are defined as small (*η*_*p*_^2^ = 0.01), medium (*η*_*p*_^2^ = 0.06), and large (*η*_*p*_^2^ = 0.14) [49].

## Results

### Collagen sources dataset

The IUNA dataset contained 2552 unique food codes, of which 28.8% (*n* = 736) were manually identified by the current investigators (CDN) to contain collagen. Food codes contained both individual food items and complete recipes. Excluding food codes that did not contain collagen protein, the collagen composition (g/100 g) of food codes in this database ranged from 0.06 g/100 g (that is, soup, chicken, and no vegetables) to 5.9 g/100 g (that is, bratwurst).

### Habitual collagen intake in Irish adults

**FIGURE 1:**
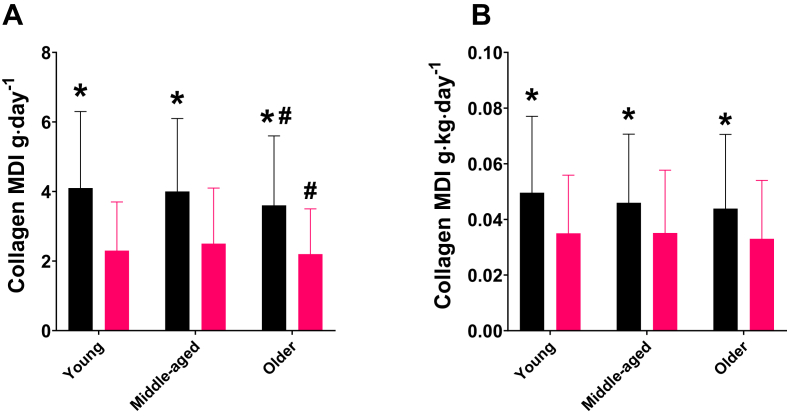
(A) Absolute and (B) normalized (to body mass) mean daily intake (MDI) of collagen in young, middle-aged, and older males (black bars) and females (pink bars). ^∗^Higher than females (*P* < 0.001) and ^#^lower than middle aged (*P* = 0.021).

Absolute and relative collagen MDI are displayed in Figure 1. The collagen MDI for the entire sample (*n* = 1338) was 3.2 ± 2.0 g/d. There was a main effect of sex on collagen MDI (*F*_1, 1331_ = 217.042, *P* < 0.001, *η*_*p*_^2^ = 0.140), where intake for males (4.0 ± 2.1 g/d; *n* = 657) was higher than for females (2.4 ± 1.4 g/d; mean difference = 1.6 g/d; *P* < 0.001). There was also a main effect of age on collagen MDI (*F*_2, 1331_ = 3.914, *P* = 0.020, *η*_*p*_^2^= 0.006). Collagen MDI was 3.2 ± 2.0 g/d, 3.3 ± 2.0 g/d, and 2.9 ± 1.8 g/d for young, middle-aged, and older adults, respectively. Post-hoc comparisons revealed a mean difference of 0.4 g/d between middle-aged and older adults (*P* = 0.021), but there were no differences between young and middle-aged (*P* = 1.000), or between young and older adults (*P* = 0.053). There was no interaction between sex and age on collagen MDI (*F*_2, 1331_ = 1.021, *P* = 0.360, *η*_*p*_^2^ = 0.002), and PA level did not influence the statistical model (*F*_1, 1331_ = 0.161, *P* = 0.689, *η*_*p*_^2^= 0.000).

With regards to relative collagen MDI (collagen intake relative to body mass), the intake for the entire sample (*n* = 1338) was 0.05 ± 0.03 g/kg/d. There was a main effect of sex on collagen MDI (*F*_1, 1331_ = 70.873, *P* < 0.001, *η*_*p*_^2^ = 0.052), with males’ intake being higher than females. Specifically, the males’ intake was 0.05 ± 0.03 g/kg/d, whereas the females’ intake was 0.03 ± 0.02 g/kg/d (mean difference = 0.01 g/kg/d; *P* < 0.001). There was no main effect of age on relative collagen MDI (*F*_2, 1283_ = 2.224, *P* = 0.108, *η*_*p*_^2^= 0.003). Intake in young adults was 0.04 ± 0.03 g/kg/d, whereas intake in both middle-aged and older adults was 0.04 ± 0.02 g/kg/d. There was no interaction between sex and age on relative collagen MDI (*F*_2, 1283_ = 0.97, *P* = 0.379, *η*_*p*_^2^ = 0.002). Additionally, PA level did not influence the model (*F*_1, 1283_ = 0.01, *P* = 0.91, *η*_*p*_^2^= 0.000).

**FIGURE 2:**
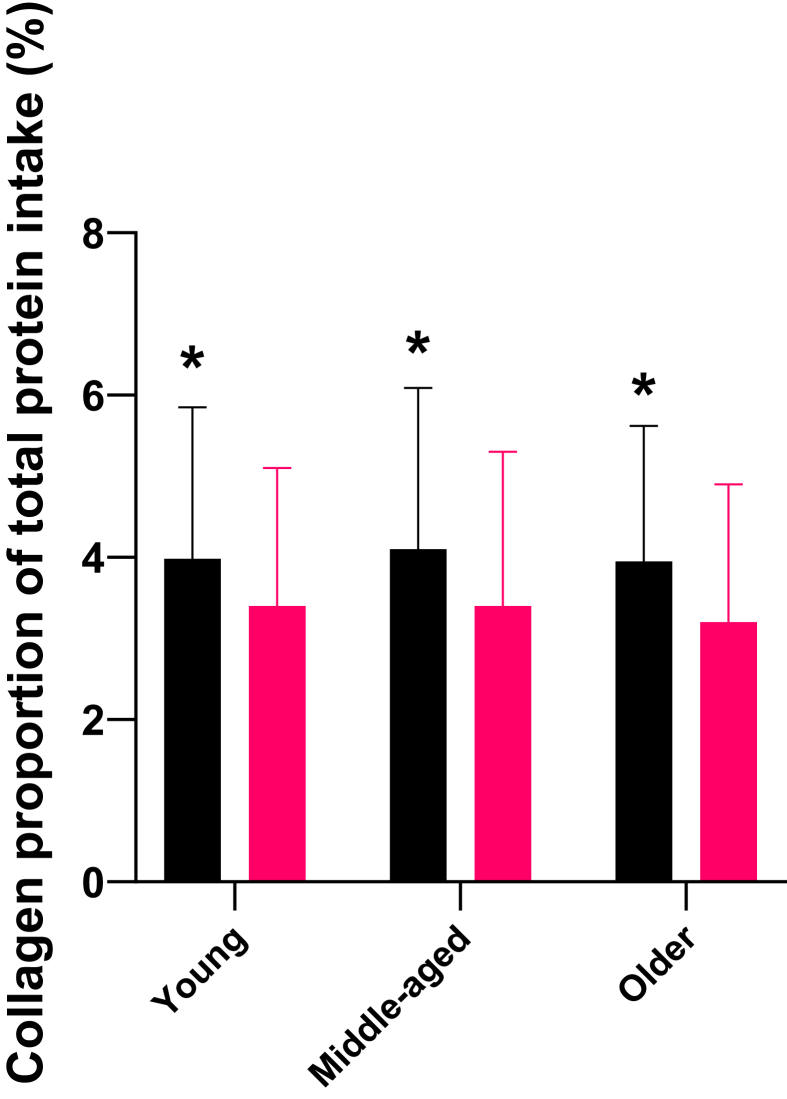
Mean daily intake (MDI) of collagen expressed as a percentage of total daily protein intake in young, middle-aged, and older males (black bars) and females (pink bars). ^∗^Higher than females (*P* < 0.001).

Collagen MDI as a percentage of total daily protein intake is displayed in Figure 2. There was no main effect of age (*F*_1,1331_ = 1.185, *P* = 0.306, *η*_*p*_^2^ = 0.001); however, males consumed a greater amount of collagen (4.0% ± 1.9%) as a proportion of total protein intake compared with females (3.4% ± 1.8%; *F*_1,1331_ = 41.359, *P* < 0.001, *η*_*p*_^2^ = 0.030), but there was no age × sex interaction (*F*_1,1331_ = 0.470, *P* = 0.625, *η*_*p*_^2^ = 0.001). These findings persisted when collagen intake and total protein intake were normalized to body mass (age group: *F*_1,1331_ = 0.977, *P* = 0.377, *η*_*p*_^2^ = 0.002; sex: *F*_1,1331_ = 40.189, *P* < 0.001), *η*_*p*_^2^ = 0.030; age × sex interaction: *F*_2,1283_ = 0.669, *P* = 0.513, *η*_*p*_^2^= 0.001).

### Energy and macronutrient intake of Irish adults

Mean daily energy and macronutrient intake are detailed in Table 1. The mean daily energy intake for the entire sample (*n* = 1338) was 2006 ± 639 kcal/d. There were main effects of age (*F*_2, 1492_ = 28.703, *P* < 0.001, *η*_*p*_^2^ = 0.032) and sex (*F*_1,1492_ = 367.756, *P* < 0.001, *η*_*p*_^2^ = 0.208) on daily energy intake, and there was an interaction between age and sex (*F*_2, 1492_ = 4.747, *P* = 0.004, *η*_*p*_^2^ = 0.005). With regard to absolute intake, males had a higher mean energy intake compared with females across all age groups. Regarding age, energy intake was lower in older adults compared with both young adults and middle-aged adults. No differences were observed in energy intake between young adults and middle-aged adults for either sex. The interaction was likely a result of the sex difference in middle-aged adults, being smaller than the sex differences in both young and older adults.

**TABLE 1:** Habitual energy, protein, carbohydrate, fat, collagen, and vitamin C intake of Irish adults.

Nutrient		Young adults (*n* = 596)	Middle-aged adults (*n* = 575)	Older adults (*n* = 167)	Effect of sex	Effect of age	Sex × age interaction
Energy intake (kcal/d)							
	All	2135 ± 692	1952 ± 576	1808 ± 555	*P* < 0.001	*P* < 0.001	*P* = 0.004
	Male	2481 ± 656	2256 ± 578	2086 ± 585			
	Female	1755 ± 506	1689 ± 425	1564 ± 391			
Energy intake (kcal/kg/d)							
	All	28.8 ± 9.3	25.2 ± 7.5	26.7 ± 8.7	*P* < 0.001	*P* < 0.001	*P* = 0.93
	Male	30.5 ± 9.5	26.1 ± 7.6	25.5 ± 8.9			
	Female	26.8 ± 8.7	24.2 ± 7.6	23.8 ± 7.3			
PRO intake (g/d)							
	All	87 ± 31	84 ± 25	79 ± 24	*P* = 0.005	*P* < 0.001	*P* < 0.001
	Male	104 ± 31	97 ± 24	91 ± 24			
	Female	69 ± 20	73 ± 20	69 ± 18			
PRO intake (g/kg/d)							
	All	1.2 ± 0.4	1.1 ± 0.3	1.1 ± 0.4	*P* < 0.001	*P* = 0.002	*P* < 0.001
	Male	1.3 ± 0.4	1.1 ± 0.3	1.1 ± 0.4			
	Female	1.0 ± 0.3	1.1 ± 0.3	1.0 ± 0.4			
CHO intake (g/d)							
	All	243 ± 82	225 ± 76	214 ± 69	*P* < 0.001	*P* < 0.001	*P* = 0.053
	Male	278 ± 84	257 ± 83	240 ± 74			
	Female	205 ± 60	197 ± 57	191 ± 54			
CHO intake (g/kg/d)							
	All	3.3 ± 1.1	2.9 ± 1.0	2.9 ± 1.0	*P* = 0.001	*P* < 0.001	*P* = 0.277
	Male	3.4 ± 1.2	3.0 ± 1.1	3.0 ± 1.0			
	Female	3.1 ± 1.0	2.9 ± 1.0	2.8 ± 1.0			
Fat intake (g/d)							
	All	81 ± 30	75 ± 27	70 ± 29	*P* = 0.001	*P* < 0.001	*P* = 0.326
	Male	92 ± 30	86 ± 29	80 ± 33			
	Female	68 ± 24	66 ± 21	61 ± 20			
Fat intake (g/kg/d)							
	All	1.3 ± 0.5	1.2 ± 0.5	1.1 ± 0.5	*P* < 0.001	*P* < 0.001	*P* = 0.445
	Male	1.4 ± 0.5	1.3 ± 0.5	1.2 ± 0.6			
	Female	1.2 ± 0.5	1.1 ± 0.4	1.0 ± 0.5			
Vitamin C intake (mg/d)							
	All	134 ± 279	119 ± 175	132 ± 215	*P* = 0.020	*P* = 0.516	*P* = 0.575
	Male	127 ± 167	104 ±130	104 ± 134			
	Female	141 ± 363	131 ± 205	141 ± 291			

Values are expressed as mean ± SD. Young adults were aged 18–39 y; middle-aged adults were aged 40–64 y; older adults were aged >65 y.

Abbreviations: CHO, carbohydrate; PRO, protein.

When normalized to body mass, the daily energy intake for the entire sample was 26.6 ± 8.7 kcal/kg/d. There were main effects of age group and sex on normalized energy intake (*F*_1,1492_ = 31.143, *P* < 0.001, *η*_*p*_^2^ = 0.022; *F*_1,1492_ = 29.501, *P* < 0.001, *η*_*p*_^2^ = 0.022) but no age × sex interaction (*F*_2, 1492_ = 2.751, *P* = 0.93, *η*_*p*_^2^ = 0.005). Normalized energy intake was lower in young compared with middle-aged adults, but there was no difference between middle-aged and older adults. Females had lower normalized energy intake compared with males in all age groups.

Regarding macronutrient intake, there were main effects of age, and sex on protein, carbohydrate, and fat intake, respectively (*P* < 0.01). However, there was only an interaction between age and sex on absolute and normalized protein intake (*F*_2, 1331_ = 9.968, *P* < 0.001, *η*_*p*_^2^ = 0.015; *F*_2, 1283_ = 8.383, *P* < 0.001, *η*_*p*_^2^ = 0.013), and not regarding any other macronutrient intake normalized to body mass. Absolute and normalized protein intake was lower in females (*P* < 0.001) but was not different across age groups. Absolute protein intake in males, however, tended to be lower with age (young compared with middle-aged, *P* = 0.006, young compared with old, *P* < 0.001, middle-aged compared with old, *P* = 0.064). Normalized protein intake in males was lower in middle-aged and older compared to young (both groups compared with young, *P* < 0.001), but there was no difference between middle-aged and old (*P* = 1.000). Although protein intake was generally consistent across most groups, young males had a notably higher intake compared with their female counterparts and other age groups.

Normalized carbohydrate intake was lower in females, and regardless of sex, intake in young adults was higher than in both middle-aged and older adults, and intake in middle-aged adults was higher than in older adults. There was a main effect of age (*F*_1,1331_ = 13.482, *P* < 0.001, *η*_*p*_^2^ = 0.020) and sex (*F*_1,1331_ = 13.482, *P* < 0.001, *η*_*p*_^2^ = 0.127) on fat intake, but no sex × age interaction (*F*_2,1331_ = 1.122, *P* = 0.127), which was similar for normalized fat intake (age: *P* < 0.001, *η*_*p*_^2^ = 0.025, sex: *P* < 0.001, *η*_*p*_^2^ = 0.001, sex × age: *P* = 0.445, *η*_*p*_^2^ = 0.001). PA was included as a covariate in the ANCOVA model to account for potential confounding effects. However, this variable did not influence the model for any nutrient apart from fat intake. PA was associated with mean daily fat intake (*F*_1,1331_ = 4.20, *P* =0.041, *η*_*p*_^2^ = 0.003) and normalized fat intake (*F*_1,1283_ = 4.65, *P* = 0.031, *η*_*p*_^2^ = 0.004), explaining 0.3%–0.4% of the variance. This indicates that, although PA levels were statistically associated with fat intake, its influence on the model was minimal. Importantly, even after adjusting for PA, main effects of sex and age on fat intake were observed.

### Habitual vitamin C intake of Irish adults

Vitamin C MDI is presented in Table 1. Age had no main effect on vitamin C MDI (*F*_2, 1331_ = 0.661, *P* = 0.516), and no age × sex interaction (*F*_2,1331_ = 0.554, *P* = 0.575). However, there was a main effect of sex (*F*_2,1331_ = 5.448, *P* = 0.020, *η*_*p*_^2^ = 0.004), where females’ intake (141 ± 291 mg/d) was higher than for males (115 ± 151 mg/d) (*P* < 0.001). PA did not influence the ANCOVA model (*F*_1,1331_ = 0.239, *P* = 0.625).

### Habitual PA levels of Irish adults

Although the NANS included 1500 respondents in its dataset, 162 cases were excluded from this study because of incomplete PA questionnaires, leaving a final 1338 valid cases used for analysis. From the final analyzed sample, 1135 cases were in the “low” PA category, with 534 (47.0%) males and 601 (53.0%) females. The age distribution of this PA subgroup was as follows: 475 (41.9%) young (231 males and 244 females), 495 (43.6%) middle-aged (226 males and 269 females), and 165 (14.5%) older (77 males and 88 females). There was no significant association between age group and sex in the "low" PA category (χ^2^(2, *n* = 1135) = 0.958, *P* = 0.619).

Similarly, there was no significant association between age group and sex in the "moderate" PA category (χ^2^(2, *n* = 184) = 2.614, *P* = 0.271). Of the 184 cases in the "moderate" PA category, 109 (59.2%) were males and 75 (40.8%) were females. The age distribution was 108 (58.7%) young (69 males and 39 females), 75 (40.8%) middle-aged (40 males and 35 females), and 1 (0.5%) older (0 males and 1 female).

In contrast, the analysis for the "high" PA category revealed a significant association between age group and sex (χ^2^(2, *n* = 19) = 8.051, *P* = 0.018). Specifically, there was a higher proportion of young adults engaged in "high" PA compared with middle-aged and older adults. Additionally, the sex distribution within the "high" PA category differed notably, with males being more represented among the younger age group compared with females. For the 19 cases in this category, there were 14 (73.7%) males and 5 (26.3%) females. The age distribution was 13 (68.4%) young (12 males and 1 female), 5 (26.3%) middle-aged (2 males and 3 females), and 1 (5.3%) older (0 males and 1 female). Finally, the aggregate data across all PA categories revealed no overall association between age group and sex (χ^2^(2, *n* = 1338) = 4.534, *P* = 0.104).

## Discussion

The main objective of this study was to provide the first estimate of collagen intake in a European adult population, based on data from the NANS. The main findings were that the collagen MDI for the entire study population was ∼3 g/d, which represented just ∼4% of all protein consumed daily. This intake is considerably lower than the doses necessary to enhance collagen synthesis in intervention studies. Specifically, in young males, 15 g but not 5 g of gelatin increased whole-body collagen synthesis after skipping exercise [16], whereas 30 g, but not 15 g of collagen hydrolysate was required to enhance collagen synthesis after resistance exercise [15]. This suggests that the levels of collagen habitually consumed in the Irish diet are likely insufficient to elicit a meaningful collagen synthesis response. Interestingly, in our study, males had greater absolute intakes of collagen than females regardless of age, and this sex difference remained when intakes were adjusted for body mass. Furthermore, older adults consumed less collagen than middle-aged adults in absolute terms (with no difference between middle-aged and young adults) but this age difference disappeared when collagen intake was normalized to body mass. Notably, habitual collagen MDI was not influenced by habitual PA levels; that is, more physically active individuals did not ingest more collagen.

The collagen MDI for the total population was remarkably low; thus, the availability of exogenous glycine, proline, and hydroxyproline (the highly abundant amino acids in collagen known to stimulate collagen synthesis [50,51]) is also limited. Glycine is not an essential amino acid, as it can be synthesized from other amino acids (predominantly serine, but threonine, choline and glyoxylate may make minor contributions) [6,52]. However, habitual protein/amino acid intake plays a role in supplying glycine, proline, and hydroxyproline to support connective tissue turnover in the skin, heart, blood vessels, and musculoskeletal tissue, although it has been shown that dietary glycine intake between 1.5 and 3 g/d falls short of the amount required for collagen synthesis in metabolism [6]. Although glycine may be available from some food sources, such as soy or legumes, the amount of glycine available from the ∼3 g MDI collagen in the Irish diet is likely to be as low as 1 g/d because glycine comprises 1/3 of collagen [53].

Moreover, collagen is the only dietary source of hydroxyproline. It has been demonstrated in human dermal fibroblasts that hydroxyproline stimulates collagen synthesis in 2 ways: *1*) by increasing transforming growth factor beta 1 levels; and *2*) by directly stimulating the protein kinase B (AKT) and mammalian target of rapamycin signaling pathways [54]. Given this crucial role in supporting connective tissue turnover, the unique presence of hydroxyproline in collagen further emphasizes the importance of including collagen in the diet.

Despite this importance, the only previously reported habitual intakes of collagen were the 3–23 g/d in ‘the standard American diet’ reported by Paul et al. [24]. There are several reasons for the discrepancies between our data and those of Paul et al. [24], where our data are much closer to the lower end of collagen MDI estimates in the study by Paul et al. (2019). There are crucial differences in methodology, where Paul et al. [24] estimated collagen MDI by averaging collagen content (% dry weight) across multiple food groups, and expressed this as a percentage of mean male and female intake at population level, as calculated in the NHANES. In contrast, our study applied specific collagen content values to 736 food items on an individual basis and integrated these into the food diaries of each participant in NANS. For example, Paul et al. [24] grouped beef, pork, veal, lamb and game, and assigned these foods a collagen content of 5.15% of product dry weight; however, the collagen content of pork cuts alone can vary from ∼1% to ∼22% [31]. Moreover, any of these foods could be included in a food mixture, which is not accounted for in these analyses by Paul et al. [24]. Finally, the higher end of collagen MDI range reported in the NHANES may be attributed to differences in regional food regulation, as European Union Regulation No. 1169/2011 sets out maximum connective tissue content (measured as collagen content) for ingredients designated by the term "meat" at 25% [31]. In stark contrast, Paul et al. [24] report the collagen content of frankfurters, sausages, and luncheon meat in to be ∼55% in the United States, highlighting the lack of applicability of these findings across different jurisdictions, and the likelihood that collagen intake is lower in Europe compared with the United States.

We found that dietary collagen intake was lower in Irish female compared to male adults, both in absolute terms and relative to both body mass and total protein intake. Using the same dataset as used in this study, Hone et al. [27] recently reported that animal-based foods contributed to a larger proportion of total energy and were the dominant source of protein intake in the Irish adult diet. However, females obtained a higher proportion of protein from plant sources compared with males [27], and because collagen is exclusively found in animal products, this may explain the lower relative collagen intake in females, despite similar relative protein intake. Although the mean difference in total collagen intake between males and females appears modest (1.6 g/d), the large effect size (ηp^2^ = 0.140) suggests that future research should explore sex-specific recommendations for increasing collagen intake, with the goal of improving connective tissue health, especially in females.

The lower daily protein intake observed in older adults aligns with findings in other Western European jurisdictions [[55], [56], [57]]. It is striking, however, that there was an age × sex interaction regarding protein, and not collagen, intake relative to body mass. There are known effects of age and sex on collagen turnover in healthy humans, especially in type I collagen, the main ECM component of bone, tendon, and ligament [[58], [59], [60]]. Biomarkers of type I collagen synthesis decline from young adulthood until middle age before leveling off in both males and females [59,60]. Moreover, young and middle-aged females display lower levels of collagen synthesis than males, with the lowest levels reported in middle-aged, premenopausal females, and an increase in the postmenopausal years [59]. These observational data suggest a role for hormonal status on collagen turnover. Consequently, middle-aged and older Irish females, in particular, who also have the lowest intake of collagen according to our data, may have different dietary collagen requirements to young adults. Additionally, study designs that seek to measure the effects of dietary collagen should avoid grouping male and female participants, as differences in collagen turnover and hormonal status, even in age-matched participants, are likely to lead to erroneous conclusions.

We estimated habitual vitamin C intake in the Irish adult population, as it is essential for the hydroxylation of proline and lysine, a crucial step in the synthesis of collagen [1]. Although vitamin C intake was ∼23% greater in females than males, our sex-specific values were similar to the recommended dietary allowance of 95 and 110 mg/d for males and females, respectively [61]. However, it remains unclear whether the timing of vitamin C ingestion (for example, coingestion with collagen-rich food or supplements) is essential to optimize collagen synthesis in response to feeding. Despite adequate daily intake, the inability of humans to store vitamin C [62] suggests that any discontinuity between intake of vitamin C and collagen could potentially limit the level of endogenous collagen production.

### PA levels

We used habitual PA as a covariate when analyzing differences in collagen intake across age groups because of the recent interest in the interaction between collagen supplementation and various types of exercise [9,17]. Although not the primary outcome of our study, it is concerning that 85% of all participants were in the low activity category, with low levels of moderate PA in middle age, and indeed that only one older adult could be classified as highly active. This aligns with international data indicating lower PA levels among older populations [63,64]. A recent meta-analysis suggested that chronic exercise with collagen ingestion can improve fat-free mass, tendon and muscle morphology, maximal strength, and recovery from damaging exercise bouts [17]. Because the current study is a secondary analysis of questionnaire-based PA, we were not able to determine whether moderate or highly active participants were engaging in resistance exercise, endurance exercise, or other activities. The observation that habitual PA was lower in middle-aged adults compared with young, and only one of the older adults were highly active, supports the notion that the combined effects of increased collagen ingestion and exercise may have the greatest benefit in terms of stimulating collagen production for maintaining or improving connective tissue health in middle-aged and older populations.

### Strengths and limitations

A key strength of this study is the application of specific collagen content values to individual food items, which allowed for a more precise estimation of collagen intake compared with a previous population-level assessment that relied on broad food group averages. Additionally, these data are derived from a demographically representative sample, allowing generalizability to the Irish adult population. Our analysis is from the Irish NANS, conducted between 2008 and 2010. Dietary habits and supplement use may have changed since then, given the growth in global sales of collagen supplements and increased research in active and athletic groups. It could be suggested that collagen intake may be increasing or will increase in the future, surpassing the most recent data available for the Irish adult population. The study achieved a response rate of 60%, which is relatively high but still leaves room for potential non-response bias. The sample was demographically representative, but specific subgroups (for example, highly active middle-aged and older adults) were small, limiting the generalizability of findings to these populations.

In conclusion, habitual intake of collagen protein was remarkably low in the diet of Irish adults and may fall short of that required for optimal collagen turnover to maintain healthy connective tissues. Because collagen ingestion with exercise may improve musculoskeletal health and function, increasing collagen ingestion may be an effective strategy for maintaining connective tissue health. Achieving effective doses between 5 and 30 g through diet alone may be challenging; therefore, supplementation may be warranted. This may be especially important for females and older adults, who typically consume less collagen than males and younger adults, according to our data.

## Author contributions

The authors’ responsibilities were as follows – CDN, RME: designed the study and analyzed the data; JW: contributed to the execution of the study and provided expert advice throughout; CDN: drafted the manuscript; and all authors: read and approved the final manuscript.

## Data availability

Data described in the manuscript, code book, and analytic code will be made available upon reasonable request.

## Funding

The NANS project and preliminary analysis were supported by funding from the Irish Government, Department of Agriculture, Food and the Marine under the “Food for Health Research Initiative” 2007–2012 and Project 13 F 542—National Nutritional Databases for Public Health and New Product Development. There was no additional funding for the secondary analysis described in this manuscript.

## Conflict of interest

The authors declare no conflict of interest.
