# Supplementary material for: Habitual Dietary Collagen Intake Is Lower in Females and Older Irish Adults Compared with Younger Males
Source: J Nutr. 2025 Mar 8;155(5):1408–16. doi: 10.1016/j.tjnut.2025.03.002 (PMC12121412; doi:10.1016/j.tjnut.2025.03.002)
Supplement: Multimedia component 1 [file mmc1.docx]

**Supplementary Table 1**. Typical percentage values for total protein, collagen, and fat in meat and animal derived food products. *VL* refers to the percentage visual lean content of meat.

| **Meat Cut** | **Protein (%)** | **Collagen (%)** | **Connective Tissue (%)** | **Fat (%)** | **Literature Source** |
| --- | --- | --- | --- | --- | --- |
| **Pork** |  |  |  |  |  |
| Lean – top quality 100VL | 19.0 | 1.00 | 5.3 | 8.9 | FSAI (2008) |
| Lean containing a small amount of visible fat and connective tissue 95VL | 18.0 | 1.40 | 7.8 | 13.8 | FSAI (2008) |
| Lean containing no major gristles 90VL | 16.5 | 2.00 | 12.1 | 19.3 | FSAI (2008) |
| Sow lean 80VL | 18.0 | 2.30 | 12.8 | 26.4 | FSAI (2008) |
| Lean trimmings (incl. hock) 80VL | 17.0 | 3.40 | 20.0 | 27.4 | FSAI (2008) |
| Lean with fat 50VL | 11.5 | 1.90 | 16.5 | 53.6 | FSAI (2008) |
| More fat than lean 40VL | 6.5 | 1.10 | 16.9 | 61.8 | FSAI (2008) |
| Coarse fatty tissue containing a little lean | 5.0 | 1.50 | 1.50 | 76.5 | FSAI (2008) |
| Pork Neck Lean | 18.6 | 1.92 | 10.3 | 11.5 | FSAI (2008) |
| Pork Neck 85VL | 16.6 | 2.24 | 13.5 | 22.2 | FSAI (2008) |
| Pork Neck 85VL including Rind | 17.4 | 3.12 | 17.9 | 21.1 | FSAI (2008) |
| Pork Hand Joint Lean | 19.4 | 2.08 | 10.7 | 8.8 | FSAI (2008) |
| Pork Hand 90VL | 17.9 | 2.64 | 14.8 | 16.8 | FSAI (2008) |
| Pork Hand 90VL including Rind | 18.9 | 3.84 | 20.3 | 16.0 | FSAI (2008) |
| Pork Loin Lean | 20.9 | 1.68 | 8.0 | 8.4 | FSAI (2008) |
| Pork Loin 85VL | 17.4 | 2.08 | 12.0 | 23.9 | FSAI (2008) |
| Pork Loin 85VL including Rind | 18.9 | 3.76 | 19.9 | 22.5 | FSAI (2008) |
| Pork Belly Lean | 19.8 | 1.84 | 9.3 | 9.9 | FSAI (2008) |
| Pork Belly 80VL | 16.4 | 2.48 | 15.1 | 25.5 | FSAI (2008) |
| Pork Belly 80VL including Rind | 17.8 | 4.16 | 23.4 | 23.8 | FSAI (2008) |
| Pork Leg Lean | 20.7 | 1.6 | 7.7 | 5.0 | FSAI (2008) |
| Pork Leg 95VL | 18.8 | 2.00 | 10.6 | 13.8 | FSAI (2008) |
| Pork Leg 95VL including Rind | 19.4 | 2.96 | 15.2 | 14.0 | FSAI (2008) |
| Pork 95VL Desinewed | 17.3 | 0.55 | 3.2 | 12.0 | FSAI (2008) |
| Back Fat | 5.1 | 3.68 | 71.8 | 78.6 | FSAI (2008) |
| Flare Fat | 3.0 | 1.80 | 60.0 | 82.6 | FSAI (2008) |
| Semi-lean rind on | 16.0 | 3.20 | 20.0 | 48.6 | FSAI (2008) |
| Rind with fat uncooked (35% fat) | 22.0 | 14.20 | 64.5 | 35.0 | FSAI (2008) |
| Rind less trimmable fat uncooked (10% fat) | 34.5 | 22.40 | 64.9 | 10.0 | FSAI (2008) |
| Rind with fat cooked (derived from A12) | 17.0 | 11.00 | 64.7 | - | FSAI (2008) |
| Gristle | 22.0 | 14.20 | 64.5 | - | FSAI (2008) |
| Masseter Muscle | 20.0 | 3.90 | 19.5 | - | FSAI (2008) |
| Diaphragm | 15.0 | 10.60 | 70.7 | - | FSAI (2008) |
| Rehydrated Drinde 95VL | 22.0 | 14.20 | 64.5 | - | FSAI (2008) |
| **Beef** |  |  |  |  |  |
| Lean – top quality 100VL | 21.0 | 1.50 | 7.1 | 8.7 | FSAI (2008) |
| Lean containing a small amount of visible fat and connective tissue 95VL | 20.0 | 3.00 | 15.0 | 12.6 | FSAI (2008) |
| Lean with a moderate amount of visible fat and connective tissue 85VL | 17.0 | 3.40 | 20.0 | 22.3 | FSAI (2008) |
| Lean with some fat 75VL | 16.0 | 4.80 | 30.0 | 30.6 | FSAI (2008) |
| More fat than lean 30VL | 10.0 | 3.00 | 30.0 | 72.5 | FSAI (2008) |
| Beef Brisket Lean | 16.3 | 2.56 | 15.7 | 27.6 | FSAI (2008) |
| Beef Brisket 75VL | 15.2 | 2.88 | 19.0 | 32.4 | FSAI (2008) |
| Beef Jacob’s Ladder Lean | 18.6 | 2.40 | 12.9 | 18.4 | FSAI (2008) |
| Beef Jacob’s Ladder 85VL | 17.8 | 2.48 | 14.0 | 22.1 | FSAI (2008) |
| Beef Fore Rib Lean | 18.3 | 2.16 | 11.8 | 20.9 | FSAI (2008) |
| Beef Fore Rib 80VL | 17.1 | 2.24 | 13.1 | 25.9 | FSAI (2008) |
| Beef Chuck Lean | 19.4 | 2.48 | 12.8 | 13.1 | FSAI (2008) |
| Beef Chuck 95VL | 18.9 | 2.64 | 14.0 | 15.8 | FSAI (2008) |
| Beef Thin Flank Lean | 18.4 | 2.32 | 12.6 | 21.1 | FSAI (2008) |
| Beef Thin Flank 80VL | 16.6 | 2.64 | 15.9 | 28.8 | FSAI (2008) |
| Beef Shin and Leg Lean | 21.8 | 3.92 | 18.0 | 6.2 | FSAI (2008) |
| Beef Shin and Leg | 21.4 | 4.72 | 22.0 | 9.9 | FSAI (2008) |
| Beef Clod and Sticking Lean | 19.2 | 2.96 | 15.4 | 14.7 | FSAI (2008) |
| Beef Clod and Sticking 90VL | 18.5 | 3.20 | 17.3 | 18.2 | FSAI (2008) |
| Beef Topside Lean | 21.8 | 1.60 | 7.4 | 6.3 | FSAI (2008) |
| Beef Topside 95VL | 20.6 | 1.84 | 8.9 | 11.6 | FSAI (2008) |
| Beef Loin Rump and Fillet Lean | 19.6 | 2.00 | 10.2 | 14.8 | FSAI (2008) |
| Beef Loin Rump and Fillet 85VL | 18.0 | 2.16 | 12.0 | 22.2 | FSAI (2008) |
| Beef Thick Flank and Silverside Lean | 20.6 | 2.48 | 12.1 | 9.6 | FSAI (2008) |
| **Poultry** |  |  |  |  | FSAI (2008) |
| Skinless Chicken Breast | 23.7 | 0.62 | 2.6 | 2.1 | FSAI (2008) |
| Skinless Chicken Leg | 19.9 | 1.84 | 9.3 | 5.2 | FSAI (2008) |
| Skinless Chicken Thigh | 19.7 | 1.12 | 5.7 | 7.1 | FSAI (2008) |
| Skinless Mixed Chicken Meat | 19.4 | 1.68 | 8.6 | 7.7 | FSAI (2008) |
| Chicken Breast with Skin | 22.1 | 1.2 | 5.4 | 6.7 | FSAI (2008) |
| Chicken Leg with Skin | 18.8 | 2.4 | 12.8 | 10.1 | FSAI (2008) |
| Chicken Thigh with Skin | 17.2 | 1.84 | 10.7 | 12.9 | FSAI (2008) |
| Mixed Chicken Meat with Skin | 16.1 | 3.44 | 21.4 | 23.2 | FSAI (2008) |
| Chicken Ground Desinewed (Fronts) | 17.1 | 0.69 | 4 | 15.6 | FSAI (2008) |
| Chicken Fat | 3 | 2 | 66.7 | - | FSAI (2008) |
| Chicken Skin | 11.8 | 5.68 | 48.3 | 44.2 | FSAI (2008) |
| Skinless Turkey Breast | 23.9 | 0.64 | 2.7 | 2 | FSAI (2008) |
| Skinless Turkey Leg | 19.6 | 1.44 | 7.4 | 6 | FSAI (2008) |
| Skinless Turkey Thigh | 19.8 | 1.12 | 5.7 | 5.7 | FSAI (2008) |
| Skinless Mixed Turkey Meat | 22.1 | 1.6 | 7.2 | 6.5 | FSAI (2008) |
| Turkey Breast with Skin | 23 | 1.04 | 4.5 | 5.4 | FSAI (2008) |
| Turkey Leg with Skin | 18.9 | 1.92 | 10.1 | 9.7 | FSAI (2008) |
| Turkey Thigh with Skin | 19.1 | 1.68 | 8.8 | 10.3 | FSAI (2008) |
| Mixed Turkey Meat with Skin | 18.4 | 2.88 | 15.6 | 17.9 | FSAI (2008) |
| Turkey Skin | 12.3 | 6.56 | 53.6 | 49.4 | FSAI (2008) |
| **Marine** |  |  |  |  |  |
| Pollock (Meat) | 21 | 1.2 | 4.5 | 2.5 | Kim et al. (2007) |
| Pollock (Skin) | 19.5 | 57.1 | 5 | 1 | Kim et al. (2007) |
| Herring (Meat) | 18 | 2.5 | 4 | 3 | McBride et al. (1960) |
| Herring (Skin) | 20 | 50 | 5 | 2 | McBride et al. (1960) |
| Sardine (Meat) | 21.8 | 1.36 | - | 2.2 | Sato et al. (1986) |
| Rainbow Trout (Meat) | 21 | 1.88 | - | 0.8 | Sato et al. (1986) |
| Sea Bass (Meat) | 21.6 | 3.52 | - | 0.1 | Sato et al. (1986) |
| Japanese Eel (Meat) | 16.1 | 7.96 | - | 15.6 | Sato et al. (1986) |
| Smooth Dogshark (Meat) | 27.9 | 8.48 | - | - | Sato et al. (1986) |
| Spiny Dogfish (Meat) | 22.9 | 4.52 | - | - | Sato et al. (1986) |
| Red Stingray (Meat) | 24.6 | 3.76 | - | - | Sato et al. (1986) |
| Conger Eel (Meat) | 18.7 | 8.76 | - | - | Sato et al. (1986) |
| Pike Conger (Meat) | 20.6 | 6.16 | - | - | Sato et al. (1986) |
| Sweetfish (Meat) | 18.8 | 3.56 | - | - | Sato et al. (1986) |
| Brook Masu Salmon (Meat) | 19.9 | 1.68 | - | - | Sato et al. (1986) |
| Argentine (Meat) | 20.3 | 1.72 | - | - | Sato et al. (1986) |
| Carp (Meat) | 18.9 | 2.4 | - | - | Sato et al. (1986) |
| Striped Mullet (Meat) | 22.3 | 4.64 | - | - | Sato et al. (1986) |
| Horse Mackerel (Meat) | 21.9 | 2.04 | - | - | Sato et al. (1986) |
| Nibbler (Meat) | 21 | 3.52 | - | - | Sato et al. (1986) |
| Red Sea Bream (Meat) | 24.9 | 2.92 | - | - | Sato et al. (1986) |
| Chub Mackerel (Meat) | 23.7 | 2 | - | - | Sato et al. (1986) |
| Devil Stinger (Meat) | 20.7 | 2.72 | - | - | Sato et al. (1986) |
| Bastard Halibut (Eye Side) | 22.4 | 5.56 | - | - | Sato et al. (1986) |
| Bastard Halibut (Blind Side) | 22.6 | 5.48 | - | - | Sato et al. (1986) |
| Mud Dab (Meat) | 24.5 | 4.32 | - | - | Sato et al. (1986) |
| Black Scraper (Meat) | 19.3 | 2.72 | - | - | Sato et al. (1986) |
| Painted Comber (Meat) | 20.3 | 0.41 | - | - | Sikorski et al. (1984) |
| Rockfish (Meat) | 21.1 | 0.64 | - | - | Sikorski et al. (1984) |
| Snapper (Meat) | 21.3 | 0.56 | - | - | Sikorski et al. (1984) |
| Bigeyed Sea Perch (Meat) | 18.7 | 0.45 | - | - | Sikorski et al. (1984) |
| Red Bream (Meat) | 18.8 | 0.36 | - | - | Sikorski et al. (1984) |
| Bermuda Catfish (Meat) | 19.1 | 0.4 | - | - | Sikorski et al. (1984) |
| Marbled Notothenia (Meat) | 16.2 | - | - | - | Sikorski et al. (1984) |
| Navaga (Meat) | 19 | 0.4 | - | - | Sikorski et al. (1984) |
| Plaice (Meat) | 13.6 | 0.3 | - | - | Sikorski et al. (1984) |
| Rock Sole (Meat) | 14.4 | 0.43 | - | - | Sikorski et al. (1984) |
| Flathead Sole (Meat) | 15.6 | 0.3 | - | - | Sikorski et al. (1984) |
| Yellowfin Sole (Meat) | 15 | 0.28 | - | - | Sikorski et al. (1984) |
| Brown Sole (Meat) | 14.4 | 0.37 | - | - | Sikorski et al. (1984) |
| Australian Pilchard (Meat) | 16.5 | 0.29 | - | - | Sikorski et al. (1984) |
| Round Scad (Meat) | 17.5 | 0.18 | - | - | Sikorski et al. (1984) |
| Atlantic Bonito (Meat) | 20.6 | 0.66 | - | - | Sikorski et al. (1984) |
| Dolphin (Meat) | 15.6 | 0.5 | - | - | Sikorski et al. (1984) |
| Yellowfin Tuna (Meat) | 18.7 | 0.51 | - | - | Sikorski et al. (1984) |
| Bigeye Tuna (Meat) | 19.1 | 0.52 | - | - | Sikorski et al. (1984) |
| Spiney Lantern Shark (Meat) | 16.6 | 0.79 | - | - | Sikorski et al. (1984) |
| Squid, Loligo vulgaris (Mantle) | - | 2.68 | - | - | Sikorski et al. (1984) |
| Squid, Todarodes pacificus (Skin) | - | 2.68 | - | - | Sikorski et al. (1984) |
| Octopus, Octopus vulgaris (Arm) | - | 1.1 | - | - | Sikorski et al. (1984) |
| Octopus, Octopus vulgaris (Skin) | - | 1.9 | - | - | Sikorski et al. (1984) |
| Abalone, Haliotis discus (Adductor Muscle) | - | 0.4 | - | - | Sikorski et al. (1984) |
| Abalone, Haliotis discus (Foot Muscle) | - | 0.8 | - | - | Sikorski et al. (1984) |
| Prawn, Penaeus indicus (Muscle) | 20.9 | 0.5 | - | - | Sivakumar et al. (1996) |
| **Offal (based on fattener pigs)** |  |  |  |  |  |
| Liver | 25.12 | 0.91 | - | 3.51 | Babicz et al. (2023) |
| Heart | 17.41 | 2.16 | - | 6.39 | Babicz et al. (2023) |
| Kidney | 16.48 | 1.83 | - | 4.9 | Babicz et al. (2023) |
| **Paté** |  |  |  |  |  |
| Goat + 10% pork belly | 18.9 | 1.34 | - | 9.7 | Teixeira et al. (2019) |
| Goat + 30% pork belly | 18.7 | 1.4 | - | 13.6 | Teixeira et al. (2019) |
| Goat + 10% olive oil | 19.2 | 1.54 | - | 11.4 | Teixeira et al. (2019) |
| Goat + 30% olive oil | 19.6 | 1.54 | - | 16.6 | Teixeira et al. (2019) |
| Sheep + 10% pork belly | 22.3 | 1.3 | - | 14 | Teixeira et al. (2019) |
| Sheep + 30% pork belly | 21.9 | 1.19 | - | 18.2 | Teixeira et al. (2019) |
| Sheep + 10% olive oil | 20.7 | 1.54 | - | 13.4 | Teixeira et al. (2019) |
| Sheep + 30% olive oil | 21 | 1.46 | - | 18 | Teixeira et al. (2019) |
| **Game** |  |  |  |  |  |
| Rabbit loin | - | - | - | 1.7 | Comes et al. (2004) |
| Pheasant (Pectoralis major) | 25.13 | 0.2 | - | 0.19 | Daszkiewicz and Janiszewski (2020) |
| **Lamb and Mutton** |  |  |  |  |  |
| Lean Lamb of the Leg and Chump | 19.4 | 1.68 | 8.7 | 10.1 | FSAI (2008) |
| Lean Lamb of the Loin and Best End Neck | 18.4 | 1.76 | 9.5 | 18 | FSAI (2008) |
| Lean Lamb of the Scrag Shoulder Middle Neck and Breast | 17.1 | 1.92 | 11.2 | 21.3 | FSAI (2008) |
| 90VL Leg and Chump | 17.9 | 1.92 | 10.7 | 17.8 | FSAI (2008) |
| 80VL Loin and Best End Neck | 16 | 1.84 | 11.5 | 29.8 | FSAI (2008) |
| 80VL Scrag Shoulder Middle Neck and Breast | 15.9 | 2 | 12.6 | 27.4 | FSAI (2008) |
| Lean Fore-quarter Mutton | 16.7 | 2 | 12 | 23.1 | FSAI (2008) |
| Lean Hind-quarter Mutton | 19.3 | 1.76 | 9.1 | 11.6 | FSAI (2008) |
| 80VL Fore-quarter Mutton | 15.4 | 2.08 | 13.5 | 29.1 | FSAI (2008) |
| 90VL Hind-quarter Mutton | 17.7 | 1.92 | 10.9 | 19.6 | FSAI (2008) |


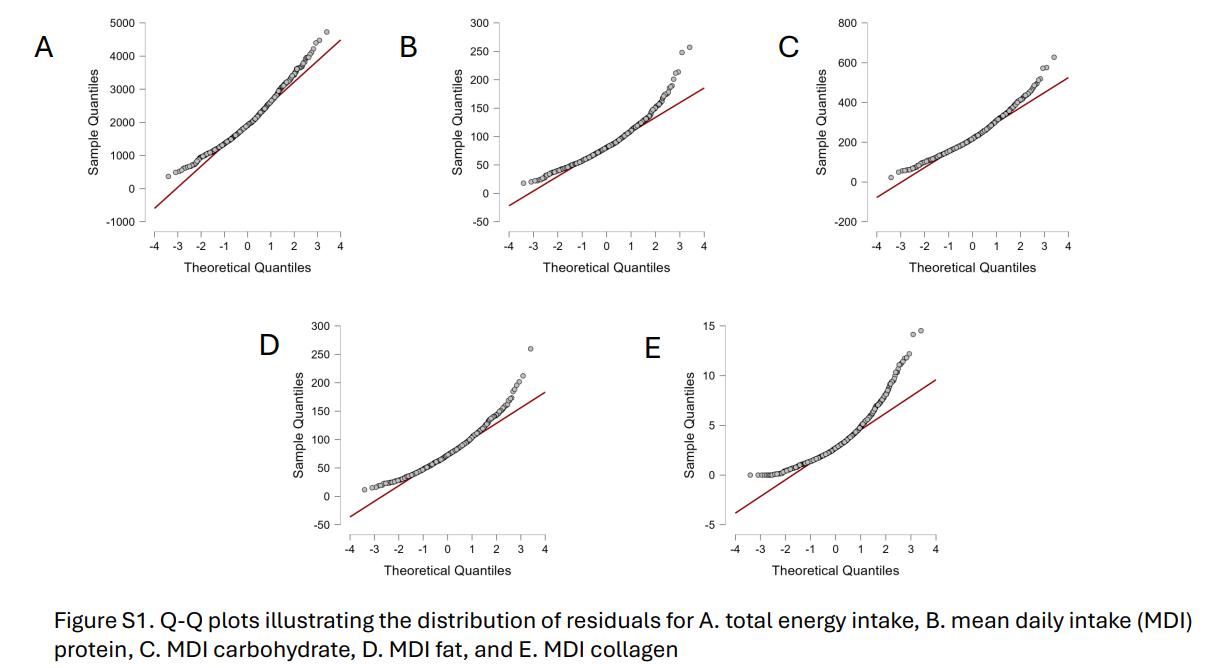


Supplementary Figure 1. Q-Q plots illustrating the distribution of residuals for A. total energy intake, B. mean daily intake (MDI) protein, C. MDI carbohydrate, D. MDI fat, and E. MDI collagen.
